# Rural Health: Low Obesity Rates Among Students in Portugal’s Countryside

**DOI:** 10.3390/nu17071153

**Published:** 2025-03-26

**Authors:** Filomena Carvalho, Sofia Silva Tavares, Radhia Aitfella Lahlou, Alexandra Varges, André R. T. S. Araújo, Cecília Fonseca, Luís R. Silva

**Affiliations:** 1 SPRINT-IPG, Sport Physical Activity and Health Research e Innovation Center, Polytechnic of Guarda, 6300-559 Guarda, Portugal; filomenacarvalho@ipg.pt (F.C.); radhialahlou@ipg.pt (R.A.L.); alexandravarges@ipg.pt (A.V.); 2 Polytechnic of Guarda, 6300-559 Guarda, Portugal; sofiasilvatavares@hotmail.com (S.S.T.); andrearaujo@ipg.pt (A.R.T.S.A.); cfonseca@ipg.pt (C.F.); 3 LAQV-REQUIMTE, Department of Chemical Sciences, Faculty of Pharmacy, University of Porto, 4050-313 Porto, Portugal; 4 CMA, Center of Mathematics and Applications, University of Beira Interior, 6201-506 Covilhã, Portugal; 5 RISE-UBI, Health Sciences Research Centre, University of Beira Interior, 6201-506 Covilhã, Portugal; 6 CERES, Department of Chemical Engineering, University of Coimbra, 3030-790 Coimbra, Portugal

**Keywords:** Body Mass Index, student health status, eating behaviours, physical activity

## Abstract

Dietary habits significantly influence students’ health status, with overweight and obesity posing serious global challenges linked to chronic diseases like type 2 diabetes and cardiovascular conditions. Our cross-sectional study assessed overweight and obesity prevalence among students in Guarda, Portugal, analyzing the nutritional and lifestyle habits of 2083 students aged 6 to 58 years. The sample included 1762 school children and 321 higher education adults, grouped into age intervals: 5–12, 13–19, 20–39, and 40–59 years. BMI analysis revealed obesity rates of 9.1% in children and 9.7% in adults, with younger children, particularly males, showing higher rates compared to older children. Increased physical activity and reduced sedentary time were correlated with a lower BMI. The observed obesity rates suggest that factors such as physical activity levels, traditional dietary patterns, and access to fresh foods in this region of Portugal may contribute to better health outcomes among students.

## 1. Introduction

The dietary and lifestyle habits of students play a fundamental role in their overall health and development [1]. There has been a marked decrease in physical activity levels among children and adolescents, partly due to increased screen time and sedentary behaviours [2,3]. Lifestyle changes among students need to be understood in order to promote early interventions to prevent obesity and reduce the risk of having chronic diseases later in life [4].

Obesity and overweight are among the most significant public health challenges facing the modern world [5,6]. Defined by the World Health Organization (WHO) as an abnormal or excessive fat accumulation that may impair health, obesity is linked to numerous non-communicable diseases (NCDs), including type 2 diabetes, cardiovascular diseases, hypertension, and certain types of cancer [5,7]. The global burden of NCDs linked to obesity continues to increase, with recent forecasts predicting that, if trends persist, over 1.31 billion people worldwide will have diabetes by 2050 [8,9]. Type 2 diabetes has been increasing among children and adolescents and can lead to severe complications such as atherosclerotic cardiovascular disease, stroke, myocardial infarction, renal insufficiency, neuropathy, vasculopathy, or retinopathy leading to blindness [10,11]. Cardiovascular diseases, including heart disease and stroke, are also more prevalent among obese individuals, contributing to higher mortality rates [12]. The incidence of cardiovascular events is expected to more than double in the next decade in some countries [13,14]. The number of obesity-related cancer cases is projected to exceed 2 million globally by 2070 [15]. Obesity is also linked to various musculoskeletal disorders, including osteoarthritis, which can cause chronic pain and disability [16]. Additionally, obesity can lead to psychological problems, such as depression, anxiety, and low self-esteem, particularly among children and adolescents [17]. Body Mass Index (BMI) is a widely recognized tool used to classify individuals’ weight categories and assess potential health risks associated with body weight. It is a simple measure derived from a person’s weight relative to their height and it is typically used to categorize weight status, identifying if someone is underweight, normal weight, overweight, or obese [18]. Overall, BMI is an essential tool in public health for monitoring population weight trends and it serves as a valuable indicator for healthcare professionals to initiate discussions about nutrition, physical activity, and lifestyle choices that contribute to overall well-being [19].

According to the WHO, in 2022, more than 2.5 billion adults were overweight, and of these, over 890 million were obese. Similarly, in the same year, 37 million children under the age of five were overweight or obese [5]. In Portugal, 10.6% of children are classified as obese, despite recent declining trends [20].

Most studies on childhood and adolescent obesity have focused on urban environments, where factors like screen time, processed food consumption, and sedentary lifestyles are well documented [21,22]. However, there is limited research on how rural environments influence student health, particularly in Portugal. To our knowledge, this is the first study examining obesity prevalence and lifestyle habits among students in a rural Portuguese setting. To address this gap, we hypothesize that students in rural areas of Portugal exhibit low obesity rates due to higher physical activity levels and more traditional and healthy dietary patterns.

## 2. Materials and Methods

**Figure 1:**
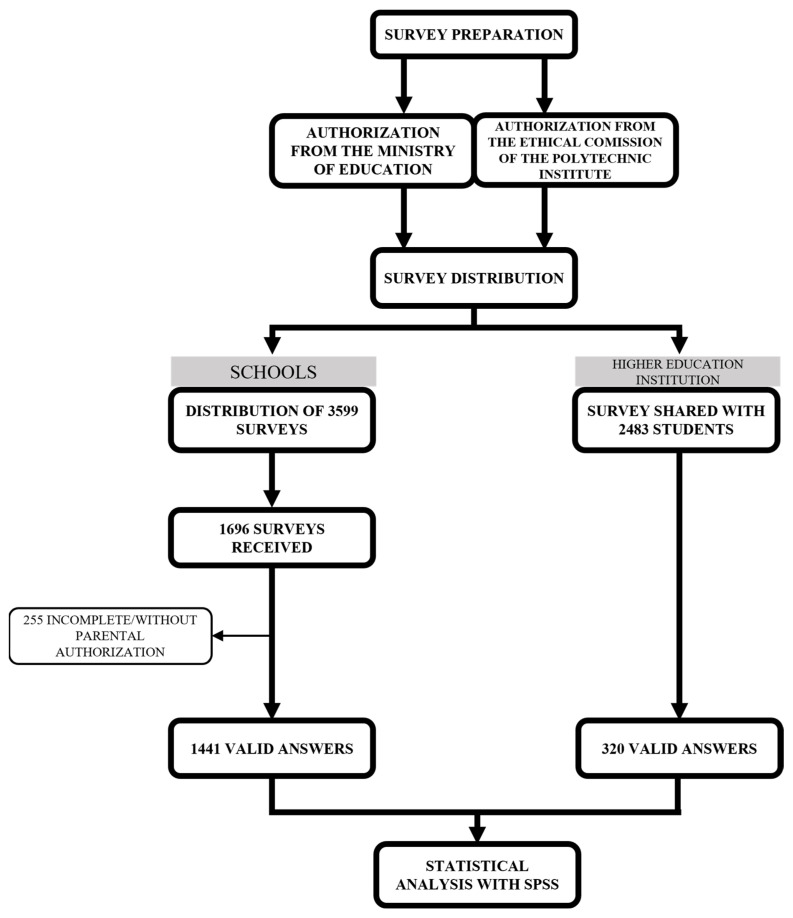
Cross-sectional study design.

To assess the nutrition and associated behaviours among students from various educational levels (1st to 12th grade and higher education), a cross-sectional study (Figure 1) was conducted from January to July of 2024. A cross-sectional design was chosen to facilitate the identification of trends and associations between lifestyle factors and health outcomes in a large sample, given the logistical limitations of conducting long-term follow-ups in a student population. The study focused on two groups of schools, namely, (1) Agrupamento de Escolas Afonso de Albuquerque and (2) Agrupamento de Escolas da Sé, and one higher education institution, (3) Instituto Politécnico da Guarda, located in Guarda, Portugal. A comprehensive survey based on national surveys performed by other universities was developed [23], which included questions related to demographics, physical activity, sleep, sedentarism, dietary habits, substance consumption, and health status (Supplementary Figure S1). These questions were designed to reflect the key aspects of nutrition that may impact student health, such as the intake of fruits, vegetables, sugary drinks, and fast food, among others, in order to understand how they correlate with health outcomes. To achieve this, students were asked to estimate the frequency with which they consume these foods. Ethical approval for this survey was obtained from the Ethical Commission of Instituto Politécnico da Guarda (Document no. 8/2024) on 21 May 2024.

A total of 3599 surveys were distributed to all the students in the two school groups, with the collaboration of the teachers. The surveys were provided in both paper and online formats, with a prior request for parental permission to participate. For younger children, parents assisted in completing the surveys at home to ensure accuracy and comprehension. Out of the 3599 distributed surveys, 1696 responses were received. A pre-test was carried out with five elements from the target population to assess question comprehension, and minor adjustments were made based on feedback. After data collection, all responses underwent a thorough validation process, where incomplete or inconsistent responses were marked and excluded. Each survey was individually reviewed, and only responses that met completeness and consistency criteria were included in the final dataset. After this process, 1441 responses were deemed valid, resulting in a response rate of 40%, since the remaining participants did not have authorization from the parents to participate or did not complete the survey in its entirety. Regarding higher education, a survey link was shared with 2483 students, through the mailing list of the institution. From this cohort, 320 students completed the survey, with a response rate of 13%. All responses to the survey were collected anonymously and kept confidential to ensure the privacy of the participants and encourage honest and accurate reporting.

The data collected were separated into the following age groups: children and adolescents (from 6 to 19 years old) and adults (over 19 years old) [5]. The study was stratified by life stages in age intervals (5–12 years, 13–19 years, 20–39 years, 40–59 years), based on commonly used classifications in public health and epidemiological research, including WHO and CDC guidelines for childhood, adolescent, and adult health assessments [24,25,26], although there is no universal definition of exact age groups. We defined overweight and obesity using the Body Mass Index (BMI) for each participant, which was calculated based on the self-reported height and weight, using the following formula [27]:

$$\mathrm{BMI}=\frac{\mathrm{weight}\;(\mathrm{kg})}{{\mathrm{height}\;(m)}^{2}}$$

BMI categories for adults were defined as follows: underweight (<18.5), normal weight (18.5–24.9), overweight (25–29.9), and obese (≥30, with further classes for obesity) (Table 1). For children and adolescents, BMI is age- and sex-specific, following WHO guidelines that use percentiles: healthy weight (15th–85th percentile), overweight (85th–97th percentile), obese (>97th percentile), thinness (3rd–15th percentile), and severe thinness (<3rd percentile) [28,29]. This approach accounts for varying growth patterns and maturity rates.

Regarding the frequency of physical activity, it was classified as low if the individual exercised less than twice per week, as moderate if three to four times per week, and heavy if more than five times per week, since the WHO recommends undertaking vigorous-intensity physical activity at least 3 days per week [31].

### Statistical Analysis

Data collected from the surveys were organized and analyzed using IBM SPSS Statistics version 29.0.0.0 for Windows. An exploratory analysis of the data was performed using frequency distributions, graphical representations, box plots, and descriptive measures (mean, median, quartiles, standard deviation, minimum, and maximum). The statistical analysis included Fisher’s exact test, chi-square tests and independent samples *t*-test to examine associations between BMI, demographic factors, dietary habits, and physical activity. When appropriate, the relative risk (RR) was used to quantify the probability of an event occurring in the exposed group compared to its probability in the non-exposed group. The Mann–Whitney U test and Spearman’s coefficient (SC) were applied for non-parametric and correlation analyses, respectively. Lastly, binary logistic regression was used to identify factors associated with BMI categories. The 95% confidence intervals (CI) were used in situations where it was considered relevant to the study. In the statistical analysis of the results, a significance level of 5% was considered.

## 3. Results and Discussion

### 3.1. Demographic Data

Regarding children and adolescents, the results included 1545 individuals, of which 43.4% were male and 56.6% were female. This group included 63.6% children aged from 6 to 12 years old and 36.4% adolescents from 13 to 19 years old. A total of 41.4% of answers were from the first cycle of education (1st to 4th grade), 17.4% belonged to the second cycle (5th to 6th grade), and 18.1% to the third cycle (7th to 9th grade). A share of 16.3% of the students were currently in high school (10th to 12th grade) and a percentage of 6.7% was enrolled in higher education.

We obtained data from a group of 217 adult students, from a higher education institution, that included 35% males and 65% females. Of the participants, 93.5% belonged to the age interval of 20 to 39 and 6.5% were 40 to 59 years old.

### 3.2. Prevalence of Overweight and Obesity Among Students

**Figure 2:**
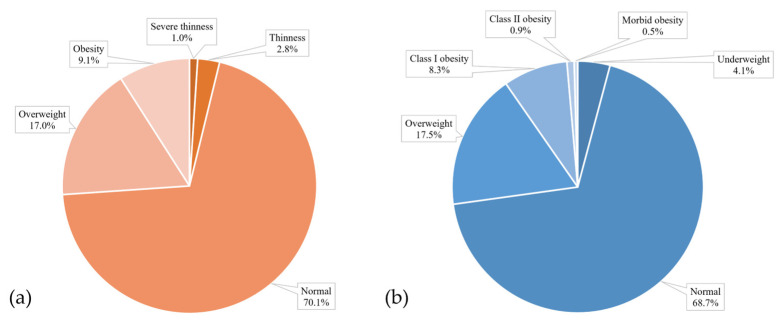
Weight status of child (**a**) and adult (**b**) students.

BMI values were calculated from self-reported height and weight data provided in the surveys. The results confirm our hypothesis, as obesity prevalence among students in Guarda was lower than national and European averages. We found that the prevalence of overweight among the children and adolescents was 17%, while 9.1% were obese (Figure 2a). A high percentage of students had a normal weight (70.1%). In Portugal, according to the COSI study, in 2021/2022, the prevalence of excess weight for children was 31.9%, from which 13.5% were obese [20]. Data from the COSI study in 33 European countries reveals a 29% prevalence of obesity in children aged 7 to 9 years old [32].

According to the data collected, 17.5% adults were overweight, while 8.3% suffered from class I obesity, 0.9% were class II obese, and 0.5% had morbid obesity. It was observed that 68.7% of adult students fell within the normal weight range (Figure 2b). As stated by the WHO European obesity report from 2022, obesity affects almost 60% of adults [24].

These findings suggest that students in Guarda, Portugal, show lower rates of obesity and overweight compared to national and European averages, indicating a potential health advantage in this region. A study has shown that children from rural areas and small cities practice more physical exercise than children from urban environments [33], which could help explain this difference.

### 3.3. Weight Status According to Sociodemographic Characteristics and Life Habits of the Students

#### 3.3.1. Age and Weight Status

A dependency was found between sex and the weight classification (*p* < 0.001), according to Fisher’s exact test. Following this information, Table 2 represents the prevalence of some characteristics and life habits in females and males aged from 6 to 19 years old, in relation to their weight status, according to BMI. In male children and adolescents, variations in BMI classification are observed across different age groups, showing a statistically significant relationship between BMI and age. Boys aged 6 to 12 showed a higher prevalence of overweight and obesity, at 33.4%, compared to boys aged 13 to 19, who showed a prevalence of 25.8% (*p* = 0.05). A similar trend is seen among female children, where 27.4% of girls aged 6 to 12 were overweight or obese, whereas this prevalence decreased to 15.0% in girls aged 13 to 19 (*p* < 0.001). Furthermore, an independent samples *t*-test confirmed that overweight/obese children tended to be younger in both male (*p* < 0.001; t(472.223) = 2.732) and female students (*p* < 0.001; t (376.01) = 5.171). Accordingly, data from the WHO in Europe 2022 reveals that one in three school-aged children are overweight or obese, while in 10–19 years the prevalence decreases to one in four [24]. These findings suggest that younger children are more susceptible to higher BMI, which is worrying, potentially due to variations in physical activity levels [34]. Younger children may not be as involved in structured physical activity as adolescents, who possibly participate in school sports and extracurricular activities more often. In fact, we found a statistically significant positive and weak relationship between age and frequency of physical activity for both sexes (female: SC = 0.095, *p* = 0.005; male: SC = 0.340, *p* < 0.001); this indicates that adolescents undertake more exercise than younger children. The characteristics of adult students were categorized similarly (Table 3). Among males aged 20–39, 30.0% were overweight/obese, compared to 24.1% of females in the same age group. The sample size for the 40–59 years old group was not relevant.

#### 3.3.2. Physical Activity and Weight Status

The frequency of weekly physical activity also shows a correlation with BMI classification in child students (male: *p* = 0.031; female: *p* = 0.002) (Table 2). The Mann–Whitney test confirmed that males with low physical activity have a higher prevalence of overweight/obesity (34.3%) compared to those with moderate (32.3%) and heavy physical activity (22.4%) (U = 42,954.5; *p* = 0.024). Similarly, females with low physical activity had a 26.3% prevalence of overweight/obesity, while those with moderate and heavy physical activity had 19.3% and 11.9%, respectively (U = 57,423.5; *p* = 0.002). This suggests that higher physical activity levels are associated with lower rates of overweight and obesity in both sexes, which is in accordance with previous studies. A systematic review of longitudinal studies found that individuals with higher physical activity levels had a significantly reduced risk of developing obesity compared to those with lower activity levels, with a lower risk of developing coronary heart disease or diabetes [35]. The Rotterdam Study, a large-scale study involving middle-aged and elderly adults, found that higher levels of physical activity significantly reduce the risk of cardiovascular disease associated with overweight and obesity [36]. The results suggest that female children that are overweight/obese spend more time sitting down during the week (SC= 0.067, *p* = 0.049), which is consistent with what was mentioned above, since spending more time sitting down logically implies less physical activity. However, among the individuals who have a low frequency of physical activity, a substantial proportion maintains a healthy weight. We need to consider the interaction with other factors, such as genetic predisposition or metabolic rates.

#### 3.3.3. Disease Incidence and Weight Status

Regarding disease incidence, there is an association between the weight status and having been diagnosed with diseases in male children (*p* = 0.035) (Table 2). According to our following tests, the incidence of health conditions among overweight or obese boys was 1.27 times higher than the incidence of health conditions among boys with a normal weight (RR = 1.27; 95% CI: 1.0–1.6). The relationship between obesity and health complications has been intensively described before. Studies show that obese children are more likely to be diagnosed with diseases such as mental disorders, gastrointestinal disorders, metabolic syndrome, insulin resistance, and non-alcoholic fatty liver disease [37,38,39]. Another study showed that children who are morbidly obese have even higher prevalence of diabetes/prediabetes and use more medications for asthma than obese children [40].

#### 3.3.4. Sleep Habits and Weight Status

There was no significant relationship between sleep and weight status. However, the descriptive data analysis suggests that the children that sleep less tend to be overweight, with 44.4% of the males who sleep less than 6 h per day being overweight or obese, compared to 30.5% who sleep 6 h or more. For females, the percentages were, respectively, 25.0% and 22.2% (Table 2). The same pattern was found in men, but not in women (Table 3). A study analyzed data from the National Health Interview Survey in the US and stated that individuals who sleep less than the recommended 7–8 h per night have significantly higher odds of being overweight or obese, and that these odds have increased in recent years. Short sleepers (5–6 h per night) had a 57% greater risk of obesity, while very short sleepers (less than 5 h per night) were twice as likely to be obese compared to those who get adequate sleep [41]. Although no significant relationship was found between sleep duration and weight, it is important to consider that we did not directly evaluate sleep quality. Research suggests that the quality of sleep plays a significant role in weight management, disrupting the metabolism and increasing appetite, which might lead to weight gain and obesity [42].

#### 3.3.5. Supplement Consumption and Weight Status

We found an association between the consumption of food supplements and the weight status in male children (*p* = 0.036) (Table 2). Further tests determined that the incidence of overweight/obesity among boys who consumed supplements was 1.4 times higher than the incidence of overweight/obesity among those who did not consume supplements (RR = 1.4; 95% CI: 1.1–1.9). A possible explanation could be the inappropriate usage of these products, coming from the parents’ erroneous assumption that the children need to take supplements to boost their growth, when in fact they do not need them. Inappropriate usage of supplements may increase a child’s calorie consumption without making up for real nutritional needs, which might result in weight gain. However, since the numbers found do not imply a direct cause-and-effect relationship, it is also possible that obese or overweight students are taking supplements to aid in weight loss, which could explain the higher incidence [43]. The consumption of supplements for memory enhancement showed there was a significant relationship with weight status in adult males (Table 3). Among male students who used this kind of supplementation, 61.5% were overweight or obese (*p* = 0.024). However, the small number of participants using memory aid supplements (13 men and 26 women) is a limitation.

#### 3.3.6. Smoking and Alcohol Consumption and Weight Status

A relationship between smoking and weight was found in women (*p* = 0.002) (Table 3). The incidence of obese woman was much higher in the smoking group (48.1%) than in the non-smoking group (18.4%). The RR of being obese/overweight in the group of women who smoke was 2.6 times the risk in the group of non-smokers (RR = 2.6; 95% CI: 1.5–4.5). Studies suggest that the relationship between smoking and obesity may vary with age and smoking intensity. Younger or heavy smokers tend to show higher rates of obesity, while older or light smokers are more likely to exhibit lower BMIs, often attributed to the appetite-suppressing effects of nicotine [44,45]. Further research with larger samples could strengthen these findings.

Alcohol consumption was significantly associated with weight status among men, with 46.9% of those who consumed alcohol being overweight/obese compared to 22.7% of non-drinkers (*p* = 0.047) (Table 3). This association suggests that alcohol consumption may be a significant risk factor for overweight and obesity. Previous research seems to lack consensus on this relationship. A review of alcohol and obesity stated that moderate alcohol consumption does not appear to be a significant risk factor for obesity, while heavy drinking and changes in alcohol consumption patterns can possibly contribute to weight gain and obesity. However, long-term effects and gender-specific responses are not yet well understood [46]. In another study of the Irish population, the individuals who drank heavily were more likely to be obese (high BMI and large waist circumference (WC)), but binge drinkers were more likely to have a larger WC and frequent alcohol consumers were less likely to be obese, suggesting a complex relationship between alcohol use and body weight [47].

**Table 3:** Characteristics and habits of adult students, categorized by weight status and separated by sex [44,45,46,47].

		Male BMI Classification (n = 76)					Female BMI Classification (n = 141)				
		Underweight/Normal Weight		Overweight/Obesity		*p*-Value	Underweight/Normal Weight		Overweight/Obesity		*p*-Value
		n	% (CI)	n	% (CI)		n	% (CI)	n	% (CI)	
Age in years	20–39	49	70.0 (58.6–79.8)	21	30.0 (20.2–41.4)	0.087	101	75.9 (68.2–82.6)	32	24.1 (17.4–31.8)	1.000
	40–58	2	33.3 (7.70–71.4)	4	66.7 (28.6–92.3)		6	75.0 (40.8–94.4)	2	25.0 (5.6–59.2)	
Frequency of weekly physical activity	Low	29	64.4 (49.9–77.2)	16	35.6 (22.8–50.1)	0.647	68	74.7 (65.1–82.8)	23	25.3 (17.2–34.9)	0.894
	Moderate	13	76.5 (53.3–91.5)	4	23.5 (8.5–46.7)		20	76.9 (58.5–89.7)	6	23.1 (10.3–41.5)	
	Heavy	9	64.3 (38.5–84.9)	5	35.7 (15.1–61.5)		19	79.2 (60.2–91.6)	5	20.8 (8.4–39.8)	
Hours of sleep per day	<6	11	64.7 (41.1–83.7)	6	35.3 (16.3–58.9)	1.000	19	82.6 (63.8–93.8)	4	17.4 (6.2–36.2)	0.595
	≥6	40	67.8 (55.2–78.6)	19	32.2 (21.4–44.8)		88	74.6 (66.2–81.8)	30	25.4 (18.2–33.8)	
Hours spent sitting down, daily, during the week	<6	24	68.6 (52.2–82.0)	11	31.4 (18.0–47.8)	1.000	42	77.8 (65.4–87.2)	12	22.2 (18.2–34.6)	0.840
	≥6	27	65.9 (50.7–78.9)	14	34.1 (21.1–49.3)		65	74.7 (64.9–82.9)	22	25.3 (17.1–35.1)	
Hours spent sitting down, daily, during the weekend	<6	36	67.9 (54.7–79.3)	17	32.1 (20.7–45.3)	1.000	65	77.4 (67.6–85.3)	19	22.6 (14.7–32.4)	0.690
	≥6	15	65.2 (44.9–82.0)	8	34.8 (18.0–55.1)		42	73.7 (61.3–83.7)	15	26.3 (16.3–38.7)	
Number of daily meals	≤3	24	68.6 (52.2–82.0)	11	31.4 (18.0–47.8)	1.000	35	67.3 (53.9–78.9)	17	32.7 (21.1–46.1)	0.102
	>3	27	65.9 (50.7–78.9)	14	34.1 (21.1–49.3)		72	80.9 (71.8–88.0)	17	19.1 (12.0–28.2)	
Smoking	Yes	13	76.5 (53.3–91.5)	4	23.5 (8.5–46.7)	0.398	14	51.9 (33.6–69.7)	13	48.1 (30.3–66.4)	0.002
	No	38	64.4 (51.7–75.7)	21	35.6 (24.3–48.3)		93	81.6 (73.7–87.9)	21	18.4 (12.1–26.3)	
Alcohol consumption	Yes	17	53.1 (36.2–69.5)	15	46.9 (30.5–63.8)	0.047	15	65.2 (44.9–82.0)	8	34.8 (18.0–55.1)	0.194
	No	34	77.3 (63.4–87.7)	10	22.7 (12.3–36.6)		92	78.0 (69.9–84.7)	26	22.0 (15.3–30.1)	
Opioids consumption	Yes	3	75.0 (28.4–97.2)	1	25.0 (2.8–71.6)	1.000	0	0	0	0	1.000
	No	48	66.7 (55.3–76.7)	24	33.3 (23.3–44.7)		107	75.9 (68.3–82.4)	34	24.1 (17.6–31.7)	
Diagnosed with disease(s)	Yes	23	65.7 (49.2–79.7)	12	34.3 (20.3–50.8)	1.000	65	75.6 (65.8–83.7)	21	24.4 (16.3–34.2)	1.000
	No	28	68.3 (53.2–80.9)	13	31.7 (19.1–46.8)		42	76.4 (64.6–86.1)	13	23.6 (13.9–36.0)	
Medication	Yes	4	50.0 (19.9–80.1)	4	50.0 (19.9–80.1)	0.427	35	71.4 (57.8–82.6)	14	28.6 (17.4–42.2)	0.411
	No	47	69.1 (57.5–79.1)	21	30.9 (20.9–42.5)		72	78.3 (69.0–85.7)	20	21.7 (14.3–31.0)	
Dietary supplementation	Yes	10	76.9 (50.3–93.0)	3	23.1 (7.0–49.7)	0.526	23	79.3 (62.2–90.9)	6	20.7 (9.1–37.8)	0.808
	No	41	65.1 (52.8–76.0)	22	34.9 (24.0–47.2)		84	75.0 (66.4–82.3)	28	25.0 (17.7–33.6)	
Memory enhancement supplementation	Yes	5	38.5 (16.5–65.0)	8	61.5 (35.0–83.5)	0.024	16	61.5 (42.4–78.2)	10	38.5 (21.8–57.6)	0.075
	No	46	73.0 (61.2–82.8)	17	27.0 (17.2–38.8)		91	79.1 (71.0–85.8)	24	20.9 (14.2–29.0)	

The findings highlight critical relationships between weight status and several factors, such as age, physical activity, and lifestyle habits. Younger children, especially boys, demonstrated higher rates of overweight and obesity. Physical activity is strongly associated with healthier BMI in both male and female children, reinforcing the need for an active lifestyle. Although correlations with supplement intake, smoking, and alcohol consumption were observed, the limited sample sizes in some categories are a limitation.

Other factors should be considered when evaluating a population’s habits and health outcomes. The socioeconomic status, for example, is one of the factors that can influence dietary habits (for example, higher consumption of more affordable energy dense foods) and access to physical activity resources, which possibly influence behaviours and, consequently, the prevalence of obesity and overweight [48]. Furthermore, psychological variables like academic stress (which can lead to depression and anxiety) may have an indirect impact on weight by influencing certain behaviours, like taking part in regular exercise, or affecting sleep [49].

It is important to sensitize parents, in an educational way, about the importance of balanced diets and regular physical activity for their children. Schools should implement programs that encourage active play and provide nutritious meals, promoting healthier habits from a young age. Furthermore, community-based programs that provide access to nutritious foods and exercise opportunities could enhance children’s health outcomes and support the fight against the rising incidence of childhood obesity.

### 3.4. Food Habits of the Student Community

**Figure 3:**
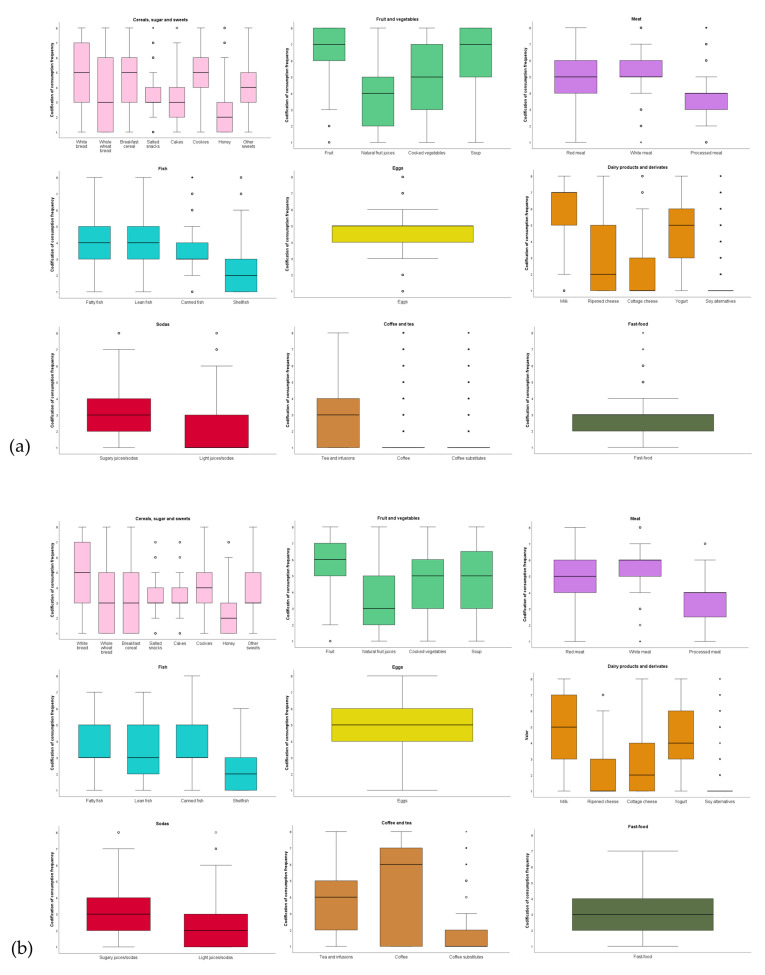
Consumption frequency of different groups of food by children (**a**) and adults (**b**). 1—never; 2—less than once per month; 3—one to three days per month; 4—one day per week; 5—two to four days per week; 6—four to six days per month; 7—once per day; 8—twice or more per day; ○ mild outlier; * extreme outlier.

We then analyzed the frequency of food consumption among children and adult students, identifying notable trends (Figure 3a,b). To assess compliance with dietary recommendations and assess overall diet quality, we used healthy and unhealthy diet indicators, based on international guidelines from WHO, World Cancer Research Fund (WCRF), and the UK Scientific Advisory Committee on Nutrition (SACN). Children frequently consumed white bread, breakfast cereals, and cookies (2–4 days/week), while whole wheat bread was less common, highlighting a missed opportunity for healthier choices. Whole grains, being a source of fiber, vitamins, minerals, phenolic compounds, and other important components, have been related to health benefits and improvement of insulin sensitivity [50]. Cakes and salted snacks were consumed moderately (1–3 days/month), likely tied to special occasions. Fruits and soups were regularly included in diets, though some children still excluded fruits. A high intake of fruits and vegetables is one indicator of a healthy diet, since the WHO recommends a daily intake of 400 g of fruits and vegetables daily to prevent noncommunicable diseases [30,51]. Adults consume slightly fewer fruits and vegetables than children, possibly due to time constraints or socioeconomic factors. Meat consumption is balanced between red and white meat (2–4 days/week). This is aligned with the recommendations by the WCRF for consuming less than 500 g of red meat per week and minimizing or avoiding processed meat [52]. The SACN advises the consumption of less than 70 g of red and processed meat per day [53]. Fish was consumed less often by adults (1–3 days/month) then by children (1 day/week). Fatty types like salmon should be consumed for their omega-3 benefits [54,55]. The SACN recommends at least two portions of fish per week, of which one should be oily [56]. Children were complying with this healthy diet indicator, while adults were not. Eggs, milk, and yogurt were staple items, with milk being consumed daily as a key source of calcium and protein, another indicator of healthy dietary habits according to European guidelines [57].

Sugary sodas and juices were consumed moderately (1–3 days/month), with some outliers. This moderation is in accordance with WHO recommendations for a reduction in the consumption of these sugars to less than 10% of the total energy intake, and, if possible, to under 5% of the total energy intake, for children and adults [58]. Fast food was consumed rarely (1–3 days/month), though some outliers showed daily consumption, raising concerns about poor dietary quality. Fast-food meals have a higher content of solid fat (23.9% of total energy) than foods from retail stores (17.6%) or schools (20.9%) [59], and their consumption is often associated with consumption of sweetened beverages and lower intake of vegetables, fruit, and milk, increasing total energy intake and leading to a poor-quality diet [60,61].

Tea and infusions were occasionally consumed, while coffee was infrequently consumed among children but common in adults (4–6 days/week). Coffee, as a major source of caffeine, is well-known for its stimulating effects that help with concentration, fatigue management, and alertness, which are particularly important for students in their demanding studying schedules. In Portugal, it also plays a central role in social and work routines, often featured in gatherings [62]. Moderate consumption has health benefits due to its content of antioxidants, which has been linked with a reduced risk of diseases such as Parkinson’s and Alzheimer’s diseases [63]. However, high caffeine intake has been associated in students with negative health effects, such as depression, anxiety, and sleep issues [64].

Statistical analysis, as detailed in Supplementary Figures S2 and S3, revealed no major associations between most food groups and weight status. However, cooked vegetables were significantly linked to healthier BMIs (*p* = 0.029 for children; *p* = 0.016 for adults), underlining their importance in daily meals. Coffee showed a significant association with higher BMI (Spearman’s correlation coefficient = 0.260; *p* < 0.001). The literature did not provide any evidence of an association between caffeine consumption and weight changes. We should consider examining whether individuals drink their coffee with sugar or not, as this could lead to an increase in daily caloric intake, which can be a possible explanation for weight gain related to coffee consumption. A study of coffee intake inversely associated an increase in the intake of unsweetened caffeinated and decaffeinated coffee with weight gain [65].

The consumption patterns observed in both children and adults suggest overall healthy dietary habits in this region and compliance with dietary guidelines. Processed and fast-food consumption appears to be relatively moderate, with most students consuming these foods occasionally. This suggests that, compared to more urbanized areas where fast food is more accessible and frequently consumed, students in Guarda may follow more traditional dietary habits, which could contribute to the lower obesity rates observed in this study. Similarly, the relatively low intake of sugary sodas and juices suggests a degree of awareness and regulation in sugar consumption. Additionally, the higher prevalence of home-cooked meals, including regular consumption of soups and vegetables, aligns with traditional Mediterranean diet patterns, which are associated with better health outcomes [66,67]. This could reflect the influence of a more rural environment, where access to fresh, local produce may be more common than in more populated cities, where processed and convenience foods dominate [68]. Moreover, lower dependence on processed meats and moderate red meat consumption indicates that families in this region may prioritize fresh, unprocessed protein sources, which is an important factor in preventing diet-related diseases [69].

### 3.5. Disease Incidence in the Student Community

Lastly, we analyzed the incidence of diseases in the student community by asking the students if they had ever been diagnosed with any disease. Firstly, we found that 66% of children had never been diagnosed with health problems before, and 34% had been diagnosed with at least one (Table 4). These results are a positive indicator of general health in the community. The most prevalent diseases found among the community of children and adolescent students were allergic (18.1%), pulmonary (8.2%), skin (7.9%), gastrointestinal (3.4%), and mental diseases (2.4%). Furthermore, we found no significant association between the diagnosis of any of the diseases and weight status (Supplementary Figures S4–S8). An association between age and the diagnosis of diseases was observed, with older children having a higher disease incidence than younger ones (U = 201,381.5; Z = −8.019; *p* < 0.001).

Regarding adult students, 55.8% had been diagnosed with one or more diseases before, while 44.2% had not. The most common diseases among this group, similarly to the children, were allergic (32.7%), pulmonary (11.1%), skin (10.1%), mental (9.2%), and gastrointestinal diseases (7.8%). In this case, we found a significant relationship between weight status and the diagnosis of pulmonary diseases (*p* = 0.03) (Supplementary Figures S9–S13). When we calculate the RR, we observed that the incidence of pulmonary diseases in the overweight/obese group is 2.3 higher than that in the underweight/normal group. A higher BMI is associated with reduced lung volume, which can manifest as a restrictive ventilatory pattern on spirometry, including reductions in expiratory reserve volume and functional residual capacity. Obese individuals may also experience decreased vital capacity, total lung capacity, and altered expiratory flow rates due to increased residual volume and airway resistance. Clinically, these patients tend to have a higher respiratory rate, leading to increased oxygen consumption [70].

An allergy epidemic has been blooming, with increased prevalence of atopic diseases such as allergic rhinitis, asthma, food allergies, conjunctivitis, and atopic dermatitis, attributed to factors like diet, the hygiene hypothesis (reduced exposure to microbes early in life), air pollution, climate change, and urbanization [71]. A cross-sectional study from primary schools in Belgium in 2019 describes a prevalence of allergic rhinitis of 29.3%, with asthma being the most significant related comorbidity [72]. According to Asher M. et al., asthma is the most frequent chronic disease among children worldwide. A systematic review from 2023 describes that prenatal and early-life exposure to traffic-related air pollution can significantly increase the risk of allergic rhinitis in children [73,74]. Although Guarda is not a highly urbanized area, environmental pollutants may still play a role, particularly considering exposure to allergens, seasonal changes, and dietary factors.

With 66% of children and 44.2% of adults reporting no previous diagnosis of disease, the overall health status of the student community appears relatively positive.

### 3.6. Logistic Regression Model

We performed a binary logistic regression model to analyze all the variables in our study in relation to BMI, separately for children and adults. The model met all prerequisites, with no multicollinearity or outliers, ensuring a sufficient number of records per variable. Using the Forward method, the analysis showed low R^2^ values (Table S1), which indicates moderate predictive capacity. This is not critical for our study, as our focus is on identifying associations rather than precise predictions. The Hosmer–Lemeshow test showed a good model fit, with significance values of 0.267 for children and 0.365 for adults, both above the 0.05 threshold, confirming that the model adequately explains the dependent variable (obesity/overweight).

In line with our previous analysis, the model presented some significant relationships. Regarding children (Table 5), the likelihood of overweight/obesity decreased with age (B = −0.137), indicating a 12.8% reduction in the odds of obesity per year (Exp(B) = 0.872). The amount of sleep was associated with a lower probability of obesity (B = −0.561). Sleeping more hours reduces the likelihood of obesity by 42.9% (Exp(B) = 0.571). A positive coefficient (B = 0.288) suggests that more time sitting down during the weekend increases the probability of obesity, with a 33.4% increase in the likelihood of obesity associated with more sitting hours during the weekend (Exp(B) = 1.334).

Lastly, females showed a 43.8% reduction in the odds of being obese compared to males (B = −0.576; Exp(B) = 0.562). In the past, data from Poland revealed a greater prevalence of obesity in boys than girls [75]. Another study with national Canadian data observed a higher prevalence of obesity in boys compared with girls aged 3–19 [76]. According to Shah et al., some biological variations in the bodies of girls and boys could explain this difference: girls tend to have greater fat mass and lower fat-free mass (e.g., muscle), which affects energy requirements, with girls requiring fewer calories than boys, thus reducing the risk of excess weight gain; girls have higher levels of leptin, a hormone that suppresses appetite and promotes energy utilization, while boys have higher levels of androgens, which suppress leptin production, leading to potentially higher appetite; and some studies suggest that brown adipose tissue, which helps burn calories, is more prevalent in females [77].

In the case of adults, the significance found for soy alternatives and coffee substitutes (Table 5) does not seem relevant, as previously we observed a low consumption of these products. A positive coefficient (B = 0.329) suggests that consuming light sodas increases the likelihood of obesity by 39% (Exp(B) = 1.390). Although these beverages contain less sugar, their consumption could potentially be associated with other unhealthy habits, such as the consumption of fast food or regularly alternating between sugary and light beverages. For coffee, we found that a higher consumption increases the likelihood of obesity by 29.5% (B = 0.258; Exp(B) = 1.295). In the case of tea and infusions, their consumption is associated with a 35.9% increase in the likelihood of obesity (B = 0.307; Exp(B) = 1.359). As we previously suggested, these associations could be related to the addition of sugar to these beverages, which needs to be further analyzed.

## 4. Limitations and Future Perspectives

Although our study provides valuable insights into the health and nutritional status of a group of students, it is important to acknowledge some limitations. First, the cross-sectional design limits our ability to establish causality between nutritional and lifestyle habits and obesity. Since the data were collected at a single point in time, we could not analyze changes over time or determine the direction of relationships. Additionally, the use of self-reported data introduced potential biases, since participants might have overestimated or underestimated their responses due to social constraints or an inaccurate memory, which may have affected the validity of our findings. Some participants, especially younger individuals, may have felt uncomfortable disclosing information about sensitive topics, such as weight, dietary habits, and consumption of substances. Furthermore, using a single measure like BMI did not allow us to fully evaluate the health status of the participants, since it does not account for body fat and muscle and, in the case of adults, for sex and age.

While the initial number of surveys was high, the rate of response was not ideal (40% for schools and 13% for higher education), which may have affected the results. Those who responded might have a greater interest in health-related topics or different lifestyle habits compared to non-respondents. Additionally, it is possible that students with overweight or obesity may have been less willing to participate in the survey due to discomfort or self-consciousness about sharing weight-related information. As a result, this could have led to an underrepresentation of students with higher BMI values, potentially influencing the estimates toward lower obesity rates. Moreover, among school students, the requirement for parental consent may have influenced participation, leading to an underrepresentation of students from households with lower parental involvement. This could have resulted in a bias toward families that are more health-conscious or engaged in their children’s education.

The fact that a part of the surveys was delivered via paper also complicated the process, since, unlike the online version, it was impossible to make the provision of answers mandatory, leading to a lack of some information and to incomplete data. Also, our study was geographically focused on students from the city of Guarda, Portugal, which may limit the applicability of our findings to other regions with different socioeconomic, cultural, and environmental contexts.

Regarding the next steps, performing longitudinal studies could be a good approach to try to establish a relationship between nutritional and lifestyle habits and obesity, identifying trends and changes over time. The accuracy of the data can be improved by using equipment like accelerometers to measure physical activity [78], and therefore obtain more objective measurements. Other geographic areas with different socioeconomic and cultural aspects should also be studied and compared. Incorporating new technologies, such as mobile health apps and online platforms, can be helpful for monitoring behaviours.

## 5. Conclusions

The findings of our study indicate a complex relationship between BMI and several factors, such as age, physical activity, sex, and dietary habits among the students. According to our survey, younger students, particularly boys, have a higher rate of overweight and obesity, which highlights the need for early intervention. Higher physical activity and reduced sedentary behaviours were associated with a healthier BMI, which emphasizes the need for promoting a more active lifestyle in schools. Despite its limitations, our study provides valuable insights into the health status of students in a non-metropolitan region, where traditional dietary habits and potentially more active lifestyles may contribute to lower obesity rates compared to national and European averages.

## Figures and Tables

**Table 1:** WHO weight classification according to BMI values [30].

BMI	Weight Classification	
<18.5	Underweight	
18.5–24.9	Normal weight	
25.0–29.9	Overweight	
30.0–34.9	Obesity class I	Obese
35.0–39.9	Obesity class II	
≥40	Obesity class III (morbid)	

**Table 2:** Characteristics and habits of children and adolescent students, categorized by weight status and separated by sex.

		Male BMI Classification (n = 670)					Female BMI Classification (n = 875)				
		Underweight/Normal Weight		Overweight/Obesity		*p*-Value	Underweight/Normal Weight		Overweight/Obesity		*p*-Value
		n	% (CI)	n	% (CI)		n	% (CI)	n	% (CI)	
Age in years	6–12	299	66.6 (62.1–70.8)	150	33.4 (29.2–37.9)	0.050	387	72.6 (68.7–76.3)	146	27.4 (23.7–31.3)	<0.001
	13–19	164	74.2 (68.2–79.6)	57	25.8 (20.4–31.8)		292	85.4 (81.3–88.8)	50	14.6 (11.2–18.7)	
Frequency of weekly physical activity	Low	188	65.7 (60.1–71.1)	98	34.3 (28.9–39.9)	0.031	368	73.7 (69.8–77.5)	131	26.3 (22.5–30.2)	0.002
	Moderate	157	67.7 (61.5–73.4)	75	32.3 (26.6–38.5)		222	80.7 (75.8–85.1)	53	19.3 (14.9–24.2)	
	Heavy	118	77.6 (70.5–83.7)	34	22.4 (16.3–29.5)		89	88.1 (80.8–93.3)	12	11.9 (6.7–19.2)	
Hours of sleep per day	<6	10	55.6 (33.2–76.3)	8	44.4 (23.7–66.8)	0.207	36	75.0 (61.5–85.5)	12	25.0 (14.5–38.5)	0.721
	≥6	453	69.5 (65.9–72.9)	199	30.5 (27.17–34.1)		643	77.8 (74.8–80.5)	184	22.2 (19.5–25.2)	
Hours spent sitting down, daily during the week	<6	150	70.1 (63.7–75.9)	64	29.9 (24.1–36.3)	0.721	181	73.3 (67.5–78.5)	66	26.7 (21.5–32.5)	0.059
	≥6	313	68,6 (64.3–72.8)	143	31.4 (27.2–35.7)		498	79.3 (76.0–82.3)	130	20.7 (17.7–24.0)	
Hours spent sitting down, daily during the weekend	<6	322	70.5 (66.2–74.5)	135	29.5 (25.5–33.8)	0.282	461	78.1 (74.5–81.2)	130	22.0 (18–8–25.5)	0.729
	≥6	141	66.2 (59.7–72.3)	72	33.8 (27.7–40.3)		218	76.8 (71.6–81.4)	66	23.2 (18.6–28.4)	
Number of daily meals	≤3	50	64.9 (53.9–74.9)	27	35.1 (25.1–46.1)	0.432	95	81.2 (73.4–87.5)	22	18.8 (12.5–26.6)	0.343
	>3	413	69.6 (65.9–73.2)	180	30.4 (26.8–34.1)		584	77.0 (74.0–79.9)	174	23.0 (20.1–26.0)	
Diagnosed with disease(s)	Yes	148	63.8 (57.5–69.8)	84	36.2 (30.2–42.5)	0.035	229	78.2 (73.2–82.6)	64	21.8 (17.4–26.8)	0.797
	No	315	71.9 (67.5–76.0)	123	28.1 (24.0–32.4)		450	77.3 (73.8–80.6)	132	22.7 (19.4–26.2)	
Medication	Yes	66	70.2 (60.5–78.7)	28	29.8 (21.3–39.5)	0.904	90	79.6 (71.5–86.3)	23	20.4 (13.7–28.5)	0.630
	No	397	68.9 (65.1–72.6)	179	31.1 (27.4–34.9)		589	77.3 (74.2–80.2)	173	22.7 (19.8–25.8)	
Dietary supplementation	Yes	45	58.4 (47.3–69.0)	32	41.6 (31.0–52.7)	0.036	66	77.6 (68.1–85.5)	19	22.4 (14.5–32.0)	1.000
	No	418	70.5 (66.7–74.1)	175	29.5 (25.9–33.3)		613	77.6 (74.6–80.4)	177	22.4 (19.6–25.4)	

**Table 4:** Incidence of diseases in children and adult students.

		Children		Adults	
		Number of Students	Percentage	Number of Students	Percentage
Diagnosed with diseases(s)	Yes	525	34.0%	121	55.8%
	No	1020	66.0%	96	44.2%
Diagnosis	Allergic diseases	279	18.1%	71	32.7%
	Pulmonary diseases	126	8.2%	24	11.1%
	Skin diseases	122	7.9%	22	10.1%
	Gastrointestinal diseases	52	3.4%	17	7.8%
	Mental diseases	37	2.4%	20	9.2%
	Heart diseases	29	1.9%	6	2.8%
	Blood diseases	26	1.7%	5	2.3%
	Kidney diseases	23	1.5%	8	3.7%
	Bone diseases	21	1.4%	5	2.3%
	Metabolic diseases	15	1.0%	7	3.2%
	Hypertension	11	0.7%	11	5.1%
	Type 1 Diabetes	5	0.3%	1	0.5%
	Dyslipidaemia	5	0.3%	3	1.4%
	Cancer	1	0.1%	1	0.5%
	Stroke	1	0.1%	2	0.9%
	Type 2 Diabetes	0	0.0%	0	0.0%

**Table 5:** Logistic regression results for significant variables in Step 5 and Step 6, for children’s and adults’ data, respectively.

		B	S.E.	Wald	*df*	Sig.	Exp(B)	9.5% CI for Exp(B)	
								Lower	Upper
Children									
Step 5	Age	−0.137	0.026	27.127	1	<0.001	0.872	0.829	0.918
	Time of sleep per day	−0.561	0.144	15.071	1	<0.001	0.571	0.430	0.758
	Hours spent sitting down, daily, during the weekend	0.288	0.090	10.369	1	0.001	1.334	1.119	1.590
	Cookies	−0.168	0.050	11.264	1	<0.001	0.845	0.767	0.933
	Sex *	−0.576	0.160	12.907	1	<0.001	0.562	0.410	0.770
	Constant	2.704	0.736	13.489	1	<0.001	14.934		
Adults									
Step 6	Cookies	0.307	0.128	5.703	1	0.017	1.359	1.057	1.748
	Soy alternatives	−0.840	0.329	6.534	1	0.011	0.432	0.227	0.822
	Light sodas	0.329	0.136	5.850	1	0.016	1.390	1.064	1.815
	Tea and infusions	0.307	0.115	7.094	1	0.008	1.359	1.084	1.702
	Coffee	0.258	0.082	9.867	1	0.002	1.295	1.102	1.521
	Coffee substitutes	−0.320	0.131	5.981	1	0.014	0.726	0.562	0.938
	Constant	−3.894	0.991	15.443	1	<0.001	0.020		

* Male/Female. B = regression coefficient; S.E. = standard error; Wald = Wald chi-square test; *df* = degrees of freedom; Sig. = significance level; Exp(B) = odds ratio; CI = confidence interval.

## Data Availability

The data presented in this study are available on request from corresponding author due to privacy.

## Supplementary Materials

The following supporting information can be downloaded at: www.mdpi.com/article/10.3390/nu17071153/s1, Figure S1: Survey for the student community; Figure S2: Comparison of consumption of different food groups by weight status of children using Mann–Whitney U test; Figure S3: Comparison of consumption of different food groups by weight status of adults using Mann–Whitney U test; Figure S4: Chi-square test results for the association between allergic diseases and BMI in children; Figure S5: Chi-square test results for the association between pulmonary diseases and BMI in children; Figure S6: Chi-square test results for the association between skin diseases and BMI in children; Figure S7: Chi-square test results for the association between gastrointestinal diseases and BMI in children; Figure S8: Chi-square test results for the association between mental diseases and BMI in children; Figure S9: Chi-square test results for the association between allergic diseases and BMI in adults; Figure S10: Chi-square test results for the association between pulmonary diseases and BMI in adults; Figure S11: Chi-square test results for the association between gastrointestinal diseases and BMI in adults; Figure S12: Chi-square test results for the association between mental diseases and BMI in adults; Figure S13: Chi-square test results for the association between skin diseases and BMI in adults; Table S1: Model summary for the logistic regression of the children’s and adults’ data.
