# Supplementary material for: Rural Health: Low Obesity Rates Among Students in Portugal’s Countryside
Source: Nutrients. 2025 Mar 26;17(7):1153. doi: 10.3390/nu17071153 (PMC11990436; doi:10.3390/nu17071153)
Supplement: Supplementary file 1 [file nutrients-17-01153-s001.zip › nutrients-3522423-supplementary.pdf]

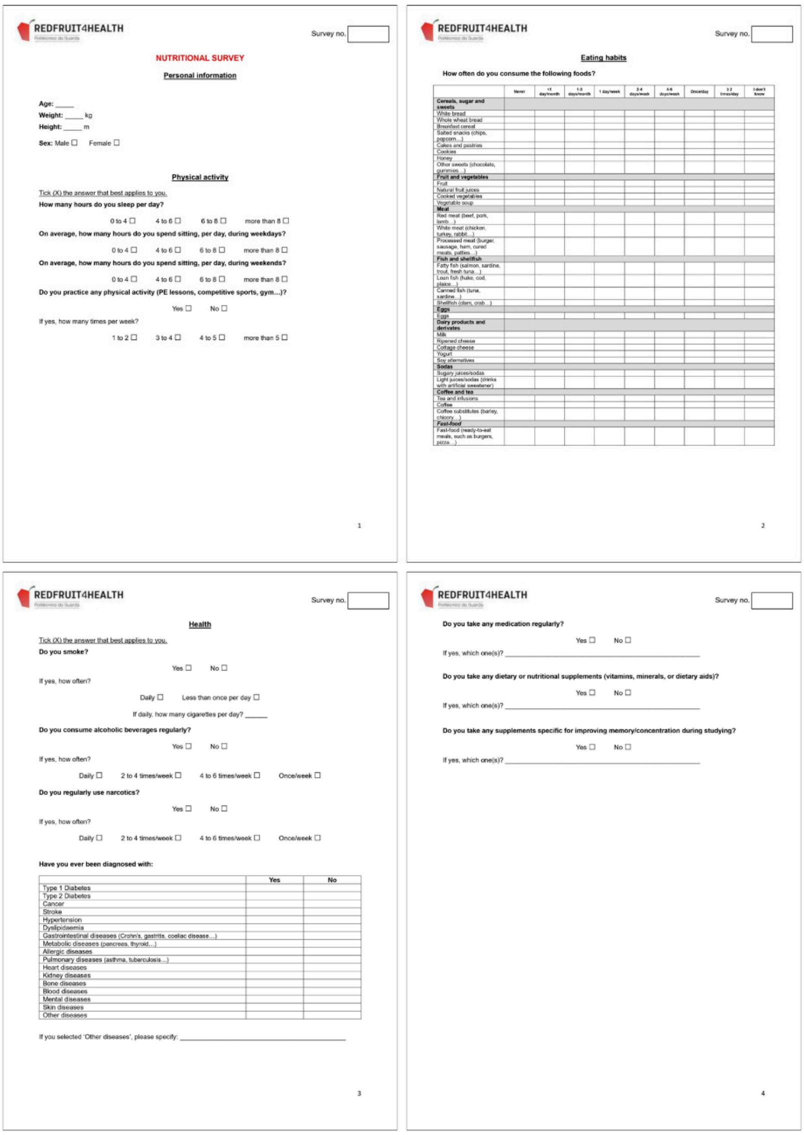

REDFRUIT4HEALTH
Politécnico da Guarda

Survey no.

NUTRITIONAL SURVEY

Personal information

Age: _____
Weight: _____ kg
Height: _____ m

Sex: Male ☐ Female ☐

Physical activity

Tick (X) the answer that best applies to you.

How many hours do you sleep per day?

0 to 4 ☐ 4 to 6 ☐ 6 to 8 ☐ more than 8 ☐

On average, how many hours do you spend sitting, per day, during weekdays?

0 to 4 ☐ 4 to 6 ☐ 6 to 8 ☐ more than 8 ☐

On average, how many hours do you spend sitting, per day, during weekends?

0 to 4 ☐ 4 to 6 ☐ 6 to 8 ☐ more than 8 ☐

Do you practice any physical activity (PE lessons, competitive sports, gym...)?

Yes ☐ No ☐

If yes, how many times per week?

1 to 2 ☐ 3 to 4 ☐ 4 to 5 ☐ more than 5 ☐

1

REDFRUIT4HEALTH
Politécnico da Guarda

Survey no.

Eating habits

How often do you consume the following foods?

	Never	<1 day/month	1-3 days/month	1 day/week	2-4 days/week	4-6 days/week	Once/day	≥2 times/day	I don't know
**Cereals, sugar and sweets**									
White bread									
Whole wheat bread									
Breakfast cereal									
Salted snacks (chips, popcorn...)									
Cakes and pastries									
Cookies									
Honey									
Other sweets (chocolate, gummies...)									
**Fruit and vegetables**									
Fruit									
Natural fruit juices									
Cooked vegetables									
Vegetable soup									
**Meat**									
Red meat (beef, pork, lamb...)									
White meat (chicken, turkey, rabbit...)									
Processed meat (burger, sausage, ham, cured meats, patties...)									
**Fish and shellfish**									
Fatty fish (salmon, sardine, trout, fresh tuna...)									
Lean fish (hake, cod, plaice...)									
Canned fish (tuna, sardine...)									
Shellfish (clam, crab...)									
**Eggs**									
Eggs									
**Dairy products and derivates**									
Milk									
Ripened cheese									
Cottage cheese									
Yogurt									
Soy alternatives									
**Sodas**									
Sugary juices/sodas									
Light juices/sodas (drinks with artificial sweetener)									
**Coffee and tea**									
Tea and infusions									
Coffee									
Coffee substitutes (barley, chicory...)									
**Fast-food**									
Fast-food (ready-to-eat meals, such as burgers, pizza...)									

2

REDFRUIT4HEALTH
Politécnico da Guarda

Survey no.

Health

Tick (X) the answer that best applies to you.

Do you smoke?

Yes ☐ No ☐

If yes, how often?

Daily ☐ Less than once per day ☐

If daily, how many cigarettes per day? ______

Do you consume alcoholic beverages regularly?

Yes ☐ No ☐

If yes, how often?

Daily ☐ 2 to 4 times/week ☐ 4 to 6 times/week ☐ Once/week ☐

Do you regularly use narcotics?

Yes ☐ No ☐

If yes, how often?

Daily ☐ 2 to 4 times/week ☐ 4 to 6 times/week ☐ Once/week ☐

Have you ever been diagnosed with:

	Yes	No
Type 1 Diabetes		
Type 2 Diabetes		
Cancer		
Stroke		
Hypertension		
Dyslipidaemia		
Gastrointestinal diseases (Crohn's, gastritis, coeliac disease...)		
Metabolic diseases (pancreas, thyroid...)		
Allergic diseases		
Pulmonary diseases (asthma, tuberculosis...)		
Heart diseases		
Kidney diseases		
Bone diseases		
Blood diseases		
Mental diseases		
Skin diseases		
Other diseases		

If you selected 'Other diseases', please specify: ______________________________

3

REDFRUIT4HEALTH
Politécnico da Guarda

Survey no.

Do you take any medication regularly?

Yes ☐ No ☐

If yes, which one(s)? ______________________________

Do you take any dietary or nutritional supplements (vitamins, minerals, or dietary aids)?

Yes ☐ No ☐

If yes, which one(s)? ______________________________

Do you take any supplements specific for improving memory/concentration during studying?

Yes ☐ No ☐

If yes, which one(s)? ______________________________

4

Figure S1. Survey for the student community.

Cereals, sugar and sweets[a]

	White bread	Whole wheat bread	Breakfast cereal	Salted snacks (chips, popcorn...)	Cakes	Cookies	Honey	Other sweets
Mann Whitney U	203031,000	186397,500	209966,000	202777,500	203151,500	193868,000	194378,000	198763,000
Wilcoxon W	275802,000	733978,500	284657,000	278243,500	278229,500	272078,000	264878,000	273068,000
Z	-,309	-1,153	-,697	-1,611	-1,525	-3,457	-1,184	-1,831
Asymp. Sig. (2-tailed)	,758	,249	,486	,107	,127	<,001	,236	,067

a. Grouping Variable: Weight status (underweight/normal weight or overweight/obese)

Fruit and vegetables[a]

	Fruit	Natural fruit juices	Cooked vegetables	Soup
Mann-Whitney U	223419,500	184171,500	197929,500	210162,500
Wilcoxon W	304020,500	251332,500	804580,500	834565,500
Z	-,432	-1,793	-2,177	-1,212
Asymp. Sig. (2-tailed)	,665	,073	,029	,226

a. Grouping Variable: Weight status (underweight/normal weight or overweight/obese)

Meat[a]

	Red meat (beef, pork, lamb...)	White meat (chicken, turkey, rabbit...)	Processed meat (burguer, sausages, ham, pastries)
Mann-Whitney U	216865,000	218535,000	206331,000
Wilcoxon W	842386,000	296745,000	282186,000
Z	-,546	-,349	-1,285
Asymp. Sig. (2-tailed)	,585	,727	,199

a. Grouping Variable: Weight status (underweight/normal weight or overweight/obese)

Fish[a]

	Fatty fish (salmon, sardine, trout, fresh tuna...)	Lean fish (hake, codfish, plaice...)	Canned fish (tuna, sardine...)	Shellfish (mussel, clam, edible crab...)
Mann-Whitney U	212942,000	214071,000	196705,000	197029,500
Wilcoxon W	288797,000	826242,000	268336,000	270949,500
Z	-,331	-,069	-1,315	-1,474
Asymp. Sig. (2-tailed)	,741	,945	,188	,140

a. Grouping Variable: Weight status (underweight/normal weight or overweight/obese)

Eggs[a]

	Eggs
Mann-Whitney U	199088,500
Wilcoxon W	793683,500
Z	-1,468
Asymp. Sig. (2-tailed)	,142

a. Grouping Variable: Weight status (underweight/normal weight or overweight/obese)

Dairy products and derivates[a]

	Milk	Ripened cheese	Cottage cheese	Yogurt	Soy alternatives
Mann-Whitney U	216321,000	194660,000	205051,000	210819,000	202585,500
Wilcoxon W	296922,000	781646,000	277441,000	824097,000	795001,500
Z	-,999	-1,805	-,143	-,771	-,196
Asymp. Sig. (2-tailed)	,318	,071	,887	,441	,845

a. Grouping Variable: Weight status (underweight/normal weight or overweight/obese)

Sodas[a]

	Sugary juices/sodas	Light juices/sodas
Mann-Whitney U	198324,000	198832,500
Wilcoxon W	271860,000	791248,500
Z	-1,913	-,555
Asymp. Sig. (2-tailed)	,056	,579

a. Grouping Variable: Weight status (underweight/normal weight or overweight/obese)

Coffee and tea[a]

	Tea and infusions	Coffee	Coffee substitutes
Mann-Whitney U	198347,500	201994,000	213803,500
Wilcoxon W	273813,500	277849,000	288881,500
Z	-2,121	-2,928	-,176
Asymp. Sig. (2-tailed)	,034	,003	,860

a. Grouping Variable: Weight status (underweight/normal weight or overweight/obese)

Fast-food[a]

	Fast-food
Mann-Whitney U	207053,500
Wilcoxon W	283298,500
Z	-1,718
Asymp. Sig. (2-tailed)	,086

a. Grouping Variable: Weight status (underweight/normal weight or overweight/obese)

Figure S2. Comparison of consumption of different food groups by weight status of children using Mann-Whitney U test.

Cereals, sugar and sweets[a]

	White bread	Whole wheat bread	Breakfast cereal	Salted snacks (chips, popcorn...)	Cakes	Cookies	Honey	Other sweets
Mann Whitney U	3922,500	3761,000	4426,000	4248,500	4348,500	4098,500	3656,500	3999,500
Wilcoxon W	5575,500	5192,000	16361,000	5959,500	6001,500	15726,500	5196,500	5539,500
Z	-,717	-,082	-,298	-,703	-,032	-,607	-1,333	-,623
Asymp. Sig. (2-tailed)	,473	,935	,766	,482	,975	,544	,183	,533

a. Grouping Variable: Weight status (underweight/normal weight or overweight/obese)

Fruit and vegetables[a]

	Fruit	Natural fruit juices	Cooked vegetables	Soup
Mann-Whitney U	4324,500	3888,000	3495,500	3590,500
Wilcoxon W	16259,500	5428,000	15276,500	14765,500
Z	-,362	-,277	-2,419	-1,369
Asymp. Sig. (2-tailed)	,717	,782	,016	,171

a. Grouping Variable: Weight status (underweight/normal weight or overweight/obese)

Meat[a]

	Red meat (beef, pork, lamb...)	White meat (chicken, turkey, rabbit...)	Processed meat (burguer, sausages, ham, pastries)
Mann-Whitney U	4193,500	3691,500	3247,000
Wilcoxon W	15518,500	15016,500	13832,000
Z	-,219	-1,204	-1,322
Asymp. Sig. (2-tailed)	,827	,229	,186

a. Grouping Variable: Weight status (underweight/normal weight or overweight/obese)

Fish[a]

	Fatty fish (salmon, sardine, trout, fresh tuna...)	Lean fish (hake, codfish, plaice...)	Canned fish (tuna, sardine...)	Shellfish (mussel, clam, edible crab...)
Mann-Whitney U	3706,000	3644,500	3856,500	3749,500
Wilcoxon W	5191,000	14084,500	5234,500	14480,500
Z	-,444	-,289	-,050	-,552
Asymp. Sig. (2-tailed)	,657	,772	,960	,581

a. Grouping Variable: Weight status (underweight/normal weight or overweight/obese)

Eggs[a]

	Eggs
Mann-Whitney U	4064,500
Wilcoxon W	15692,500
Z	-,309
Asymp. Sig. (2-tailed)	,757

a. Grouping Variable: Weight status (underweight/normal weight or overweight/obese)

Dairy products and derivates[a]

	Milk	Ripened cheese	Cottage cheese	Yogurt	Soy alternatives
Mann-Whitney U	4352,000	3307,500	3779,500	4129,500	3593,000
Wilcoxon W	6063,000	4792,500	5375,500	15454,500	5078,000
Z	-,363	-1,905	-1,032	-,383	-2,171
Asymp. Sig. (2-tailed)	,716	,057	,302	,702	,030

a. Grouping Variable: Weight status (underweight/normal weight or overweight/obese)

Sodas[a]

	Sugary juices/sodas	Light juices/sodas
Mann-Whitney U	4016,000	3375,500
Wilcoxon W	5727,000	14253,500
Z	-,944	-1,694
Asymp. Sig. (2-tailed)	,345	,090

a. Grouping Variable: Weight status (underweight/normal weight or overweight/obese)

Coffee and tea[a]

	Tea and infusions	Coffee	Coffee substitutes
Mann-Whitney U	3923,500	2827,000	3859,000
Wilcoxon W	15098,500	14303,000	5512,000
Z	-1,039	-4,067	-1,349
Asymp. Sig. (2-tailed)	,299	<,001	,177

a. Grouping Variable: Weight status (underweight/normal weight or overweight/obese)

Fast-food[a]

	Fast-food
Mann-Whitney U	4258,000
Wilcoxon W	16504,000
Z	-,695
Asymp. Sig. (2-tailed)	,487

a. Grouping Variable: Weight status (underweight/normal weight or overweight/obese)

Figure S3. Comparison of consumption of different food groups by weight status of adults using Mann-Whitney U test.

Chi-Square Tests

	Value	df	Asymptotic Significance (2-sided)	Exact Sig. (2-sided)	Exact Sig. (1-sided)
Pearson Chi-Square	,071[a]	1	,789		
Continuity Correction[b]	,037	1	,848		
Likelihood Ratio	,072	1	,789		
Fisher's Exact Test				,822	,427
Linear-by-Linear Association	,071	1	,789		
N of Valid Cases	1545				

a. 0 cells (0,0%) have expected count less than 5. The minimum expected count is 72,77.

b. Computed only for a 2x2 table

Figure S4. Chi-Square test results for the association between allergic diseases and BMI in children.

Chi-Square Tests

	Value	df	Asymptotic Significance (2-sided)	Exact Sig. (2-sided)	Exact Sig. (1-sided)
Pearson Chi-Square	1,686[a]	1	,194		
Continuity Correction[b]	1,423	1	,233		
Likelihood Ratio	1,631	1	,202		
Fisher's Exact Test				,204	,117
Linear-by-Linear Association	1,685	1	,194		
N of Valid Cases	1545				

a. 0 cells (0,0%) have expected count less than 5. The minimum expected count is 32,87.

b. Computed only for a 2x2 table

Figure S5. Chi-Square test results for the association between pulmonary diseases and BMI in children.

Chi-Square Tests

	Value	df	Asymptotic Significance (2-sided)	Exact Sig. (2-sided)	Exact Sig. (1-sided)
Pearson Chi-Square	,466[a]	1	,495		
Continuity Correction[b]	,331	1	,565		
Likelihood Ratio	,457	1	,499		
Fisher's Exact Test				,519	,279
Linear-by-Linear Association	,466	1	,495		
N of Valid Cases	1545				

a. 0 cells (0,0%) have expected count less than 5. The minimum expected count is 31,82.

b. Computed only for a 2x2 table

Figure S6. Chi-Square test results for the association between skin diseases and BMI in children.

Chi-Square Tests

	Value	df	Asymptotic Significance (2-sided)	Exact Sig. (2-sided)	Exact Sig. (1-sided)
Pearson Chi-Square	,020[a]	1	,889		
Continuity Correction[b]	,000	1	1,000		
Likelihood Ratio	,020	1	,889		
Fisher's Exact Test				,873	,499
Linear-by-Linear Association	,020	1	,889		
N of Valid Cases	1545				

a. 0 cells (0,0%) have expected count less than 5. The minimum expected count is 13,56.

b. Computed only for a 2x2 table

Figure S7. Chi-Square test results for the association between gastrointestinal diseases and BMI in children.

Chi-Square Tests

	Value	df	Asymptotic Significance (2-sided)	Exact Sig. (2-sided)	Exact Sig. (1-sided)
Pearson Chi-Square	,017[a]	1	,895		
Continuity Correction[b]	,000	1	1,000		
Likelihood Ratio	,017	1	,895		
Fisher's Exact Test				,852	,511
Linear-by-Linear Association	,017	1	,895		
N of Valid Cases	1545				

a. 0 cells (0,0%) have expected count less than 5. The minimum expected count is 9,65.

b. Computed only for a 2x2 table

Figure S8. Chi-Square test results for the association between mental diseases and BMI in children.

Chi-Square Tests

	Value	df	Asymptotic Significance (2-sided)	Exact Sig. (2-sided)	Exact Sig. (1-sided)
Pearson Chi-Square	,180[a]	1	,672		
Continuity Correction[b]	,068	1	,794		
Likelihood Ratio	,181	1	,670		
Fisher's Exact Test				,746	,400
Linear-by-Linear Association	,179	1	,672		
N of Valid Cases	217				

a. 0 cells (0,0%) have expected count less than 5. The minimum expected count is 19,30.

b. Computed only for a 2x2 table

Figure S9. Chi-Square test results for the association between allergic diseases and BMI in adults.

Chi-Square Tests

	Value	df	Asymptotic Significance (2-sided)	Exact Sig. (2-sided)	Exact Sig. (1-sided)
Pearson Chi-Square	4,738[a]	1	,029		
Continuity Correction[b]	3,739	1	,053		
Likelihood Ratio	4,331	1	,037		
Fisher's Exact Test				,049	,030
Linear-by-Linear Association	4,716	1	,030		
N of Valid Cases	217				

a. 0 cells (0,0%) have expected count less than 5. The minimum expected count is 6,53.

b. Computed only for a 2x2 table

Figure S10. Chi-Square test results for the association between pulmonary diseases and BMI in adults.

Chi-Square Tests

	Value	df	Asymptotic Significance (2-sided)	Exact Sig. (2-sided)	Exact Sig. (1-sided)
Pearson Chi-Square	,125[a]	1	,724		
Continuity Correction[b]	,005	1	,945		
Likelihood Ratio	,128	1	,720		
Fisher's Exact Test				1,000	,487
Linear-by-Linear Association	,124	1	,725		
N of Valid Cases	217				

a. 1 cells (25,0%) have expected count less than 5. The minimum expected count is 4,62.

b. Computed only for a 2x2 table

Figure S11. Chi-Square test results for the association between gastrointestinal diseases and BMI in adults.

Chi-Square Tests

	Value	df	Asymptotic Significance (2-sided)	Exact Sig. (2-sided)	Exact Sig. (1-sided)
Pearson Chi-Square	,679[a]	1	,410		
Continuity Correction[b]	,314	1	,575		
Likelihood Ratio	,648	1	,421		
Fisher's Exact Test				,433	,280
Linear-by-Linear Association	,676	1	,411		
N of Valid Cases	217				

a. 0 cells (0,0%) have expected count less than 5. The minimum expected count is 5,44.

b. Computed only for a 2x2 table

Figure S12. Chi-Square test results for the association between mental diseases and BMI in adults.

Chi-Square Tests

	Value	df	Asymptotic Significance (2-sided)	Exact Sig. (2-sided)	Exact Sig. (1-sided)
Pearson Chi-Square	,000[a]	1	,993		
Continuity Correction[b]	,000	1	1,000		
Likelihood Ratio	,000	1	,993		
Fisher's Exact Test				1,000	,584
Linear-by-Linear Association	,000	1	,993		
N of Valid Cases	217				

a. 0 cells (0,0%) have expected count less than 5. The minimum expected count is 5,98.

b. Computed only for a 2x2 table

Figure S13. Chi-Square test results for the association between skin diseases and BMI in adults.

Table S1. Model summary for the logistic regression of the children and adults' data.

	Step	-2 Log likelihood	Cox & Snell R Square	Nagelkerke R Square
**Children's model**	5	976.492	0.063	0.096
**Adults' model**	6	134.097	0.230	0.334
